# The Association Between Vitamin D Serum Level and COVID-19 Patients’ Outcomes in a Tertiary Center in Saudi Arabia: A Retrospective Cohort Study

**DOI:** 10.7759/cureus.26266

**Published:** 2022-06-23

**Authors:** Mahmoud A Alzahrani, Faisal Almalki, Ayman Aljohani, Bassam Alharbi, Bandar Alsulami, Ahmed Alhaddad, Alaa Althubaiti, Bader Khawaji, Fayssal Farahat

**Affiliations:** 1 Specialized Polyclinic Primary Health Care Department, National Guard Health Affairs (NGHA), Jeddah, SAU; 2 College of Medicine, King Saud Bin Abdulaziz University for Health Sciences College of Medicine, Jeddah, SAU; 3 College of Medicine, King Saud Bin Abdulaziz University for Health Sciences, Jeddah, SAU; 4 Basic Medical Sciences, College of Medicine, King Saud Bin Abdulaziz University for Health Sciences College of Medicine, Jeddah, SAU; 5 Infection Prevention and Control, King Abdulaziz Medical City, Riyadh, SAU

**Keywords:** severe covid-19, family medicine, infectious disease, vitamin-d deficiency, covid 19

## Abstract

Vitamin D deficiency has been associated with the risk for immune-mediated inflammatory reactions in various respiratory infections. Our study investigated the association between vitamin D deficiency and coronavirus disease 2019 (COVID-19) patients’ outcomes. We included 545 patients who were admitted to a tertiary center in Jeddah, Saudi Arabia from March 2020 to July 2021 with a vitamin D serum test result at the time of infection or prior to disease onset. The data were extracted retrospectively using a data collection sheet. Our primary outcomes were intensive care unit (ICU) admission and in-hospital mortality. The cut-off values for vitamin D were <25, 25-49, and 50-250 for deficient, suboptimal, and optimal levels respectively. Our result revealed that there is no association between vitamin D serum levels deficiency and ICU admission (OR=1.08, p=0.75) or in-hospital mortality (OR=1.74, p=0.97). ICU admission and in-hospital mortality percentages in patients with vitamin D deficiency were 14.1% and 6.4%, respectively. In comparison, percentages for patients with optimal levels were 16.67% and 6.15% for ICU admission and in-hospital mortality, respectively. Smoking was not associated with ICU admission (p=0.05) or in-hospital mortality (p=0.38). Our study does not support a relationship between vitamin D deficiency and COVID-19 patients’ outcomes. Future studies should be directed toward conducting randomized clinical trials to determine whether vitamin D has an effective role in reducing COVID-19 severity.

## Introduction

There are known functions regarding the hormonal actions of vitamin D. For example, it has vital impacts on the homeostasis of calcium and phosphate, and it is involved in bone development and remodeling. During the last two decades, emerging studies suggest that vitamin D receptors (VDR) and the vitamin D activating enzyme 1-α- hydroxylase (CYP27B1) are not exclusive to mineral metabolism. Remarkably, these receptors are expressed in immune system cells [1]. Due to the presence of vitamin D receptor (VDR) on both B and T immune cells, vitamin D plays a vital role in regulating both innate and adaptive immune systems; moreover, it promotes anti-inflammatory responses [2]. The level of vitamin D deficiency has been established to be 25 (OH) D <20 ng/ml [3]. Recent studies have revealed a strong relationship between vitamin D deficiency and autoimmune, cardiovascular, and other chronic disorders. In addition, inverse relationship between serum vitamin D levels and inflammatory markers, as vitamin D-deficient individuals experience over-expressed inflammatory markers [4]. In a study involving Irish adults, abnormally elevated inflammatory markers are associated with low vitamin D levels [5]. Another study conducted at Nerima-Hikarigaoka Hospital suggests that low serum vitamin D levels are linked to severe acute lower respiratory infection (ALRI). Other studies suggest a direct linkage between vitamin D deficiency and respiratory syncytial virus (RSV)-induced bronchiolitis. Therefore, normal serum vitamin D levels are crucial to express optimal immune responses, especially when encountering respiratory infection [6-7]. Some epidemiological studies were conducted to assess vitamin D deficiency in Saudi Arabia. A meta-analysis study suggests that the prevalence of vitamin D deficiency represented 60% of the population in Saudi Arabia between 2008 and 2015 [8]. Another systematic review conducted in Saudi Arabia that included children, adults, pregnant/lactating women, and newborns revealed that 81% of the population were vitamin D deficient [9]. Therefore, it is critical to investigate the linkage between vitamin D levels and COVID-19 severity since numerous factors influence vitamin D bioavailability. Obesity, age, ethnicity, and even cultural reasons are deemed examples of the determining factors [10].

Severe acute respiratory syndrome coronavirus 2 (SARS-CoV-2) has been a reason for infection and cause of death in millions and has led to adverse socio-economic effects [11-12]. Thus, it is critical to implement efficient therapeutic approaches and understand the risk factors to enhance patient outcomes. There are some determinants of COVID-19 severity. Gender and age represent two examples of these determinants. Noticeably, men experienced more severe outcomes compared to female patients. Moreover, patients older than 65 years are more prone to developing severe manifestations that require intensive care. These outcomes are attributed to differences and variations in type I Interferon (IFN) responses [13-14]. At the time of infection, the adaptive immune system becomes over-activated, which leads to a storm of over-expressed cytokines and C-reactive proteins. As a result, the viral load reaches an elevated level which subjects patients to fatal complications, such as pneumonia and heart failure. COVID-19 patients experience a ranging severity based on their immune system conditions. Vitamin D adjusts innate immunity by modulating the expression of pattern recognition receptors (PRR), cytokines, and interferon regulatory factors. Thus, vitamin D induces antimicrobial pathways and promotes antiviral effectors, defending the body from infectious agents and noxious intruders [15].

While some studies revealed that vitamin D serum levels are significantly associated with COVID-19 outcomes, multiple studies did not demonstrate an association between vitamin D and the severity of COVID-19 [16-17]. Due to the controversial conclusions, this study aims to determine the proportion of serum vitamin D deficiency among COVID-19 patients and to identify the association between serum vitamin D levels and COVID-19 outcomes; moreover, to assess disease outcomes among COVID-19 patients in King Abdulaziz Medical City in Jeddah (KAMC-Jeddah).

## Materials and methods

Study design, area, and settings

This is a retrospective cohort study in which the association between vitamin D serum levels and COVID-19 patients’ outcomes was investigated. The research team recorded the vitamin D serum levels in patients admitted to the hospital or required ICU admission and even death cases. The study design was retrospective cohort because the investigators conceived the study and began identifying and enrolling subjects after outcomes had already occurred. After receiving the IRB approval, the data has been collected in medical records through the Bestcare system of the National Guard Health Affairs (NGHA) in King Abdulaziz Medical City in Jeddah (KAMC - J).

Study population

All COVID-19 patients from 19/03/2020 until 8/07/2021 were reviewed. Out of 1554 COVID-19 patients, we identified 545 patients (population size) who met the inclusion criteria and were not excluded. The subject of the study included all PCR-confirmed COVID-19 patients, the age of 18 and above patients of both genders, and patients with a vitamin D result 12 months before infection. The subject of the study excluded pregnant patients, cancer, and immunocompromised patients.

Clinical data

The data collection sheet was divided into four parts. The first part included age, height, weight, body mass index (BMI), sex, vitamin D level, and the time interval of the vitamin D test. The second part consisted of information regarding the medical history: smoking, vaccination (influenza or COVID-19), and any pre-existing illnesses. The third part included the presenting history (symptoms, prognostic factors, site of admission, length of hospitalization, and final patient status). Finally, the last part included the laboratory results which were obtained at the time of admission (white blood cell count, hemoglobin, platelets count, alanine transaminase, aspartate transaminase, procalcitonin, C-reactive protein, ferritin, D-dimer, prothrombin test, partial prothrombin test, international normalized ratio test, and COVID-19 virus qualitative cycle threshold value). Regarding the vaccination of COVID-19, there were 68 patients with missing information, so they were contacted by phone number by the research team to complete the missing information.

Statistical analysis

The cut-off values for vitamin D were <25 ng/mL for deficient patients, 25-49 ng/mL suboptimal levels, and 50-250 ng/mL for optimal levels. Descriptive statistics (median and interquartile range of 25(OH)D variables, mean and standard deviation for continuous variables such as age, BMI) are reported. Fisher’s exact test and Chi-square test were used to assess group differences. Analysis of variance and independent samples t-test was used to evaluate the association. A multivariable logistic regression model was used. The odds ratio with 95% confidence intervals (CI) was calculated. Age and BMI were included as covariates in the model. Statistical tests were two-sided, and a p-value < 0.05 was considered statistically significant. Statistical analyses were performed using the JMP 14.0 software (SAS Institute Inc., Cary, NC, USA).

## Results

**Figure 1:**
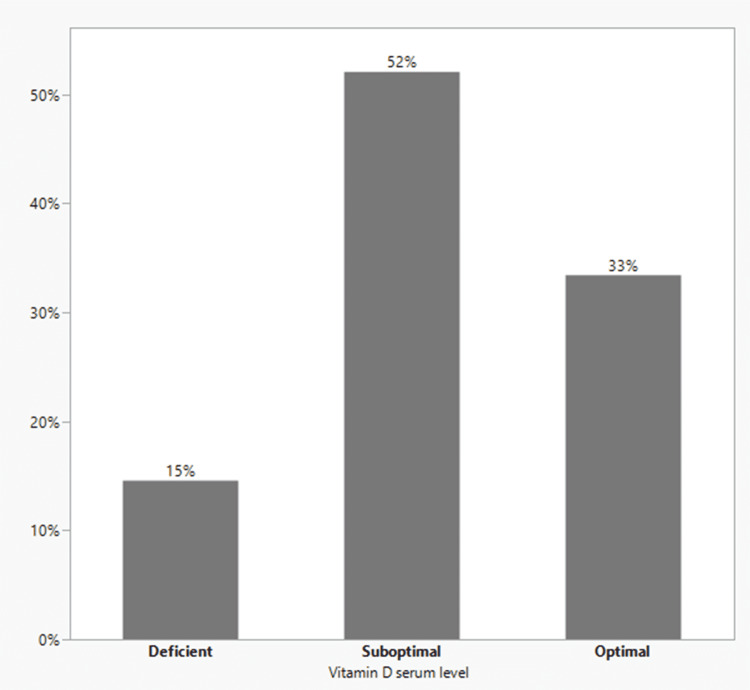
Distribution of admitted COVID-19 patients among vitamin D levels.

After applying the exclusion criteria, a total of 536 patients met our inclusion criteria. Patients’ age ranged from 19 to 95 years (mean age 54.3±16.6 years). Overall, the incidence of in-hospital mortality and ICU admission among the patients is 5.97% (n=32) and 15.1% (n=81), respectively. One patient had a vitamin D level above 250, and eight patients were home isolated and not hospital or ICU admitted, thus, a total number of nine patients were excluded from further analysis. The median of vitamin D levels is 48.6 (Q3=58.5, Q1=29.8), and the range is from 14.2 to 180.9. The prevalence of vitamin D deficiency was 14.6% (Figure 1).

Regarding characteristics among different vitamin D levels subgroups, there was an association between vitamin D levels and vomiting besides the presence of co-morbidities, specifically diabetes, hypertension, and dyslipidemia. The most prevalent symptoms among patients were cough (344, 64.2%), fever (293, 54.7%), and shortness of breath (260, 48.5%) (Table 1). Age was significantly associated with vitamin D levels (p<0.0001). Post-hoc analysis shows that mean age was significantly higher in patients with optimal vitamin D compared to patients with suboptimal or deficient vitamin D (p-value<0.0001). BMI was not significantly associated with vitamin D and COVID-19 outcomes.

**Table 1:** Characteristics of patients among different vitamin D levels. n (%) are reported unless otherwise stated. COPD: Chronic obstructive pulmonary disease

Variables	Total (n=536)	Deficient (n=78)	Suboptimal (n=279)	Optimal (n=179)	P-value
Age (years), mean±SD	54.3±16.6	49.8±18	52.4±16.4	59.1±15.3	<0.0001
Sex (Male)	286 (53.4)	35 (55.1)	161 (57.7)	90 (50.2)	0.07
BMI (kg/m^2^), mean±sd	30.5±7.0	30.5±7.3	30.0±6.5	31.3±7.4	0.14
Co-morbidities	391 (72.9)	52 (66.7)	186 (66.7)	153 (85.5)	<0.001
Diabetes	241 (44.9)	30 (38.5)	114 (40.9)	97 (54.2)	0.009
Hypertension	241 (44.9)	30 (38.5)	115 (41.2)	96 (53.6)	0.015
Dyslipidemia	117 (21.8)	9 (11.5)	59 (21.2)	49 (27.3)	0.017
Asthma	45 (8.4)	6 (7.69)	19 (6.8)	20 (11.1)	0.25
COPD	5 (0.9)	1 (1.28)	2 (0.72)	2 (1.1)	0.85
Presence of symptoms	480 (89.6)	71 (91)	254 (91)	155 (86.6)	0.28
Cough	344 (64.2)	52 (66.7)	176 (63)	116 (64.8)	0.82
Fever	293 (54.7)	44 (56.4)	154 (55.2)	95 (53.0)	0.85
Sore throat	85 (15.9)	15 (19.2)	47 (16.8)	23 (12.8)	0.35
Shortness of breath	260 (48.5)	40 (51.2)	125 (44.8)	95 (53)	0.19
Fatigue	107 (20)	21 (26.9)	54 (19.3)	32 (17.8)	0.23
Diarrhea	116 (21.6)	17 (21.8)	60 (21.5)	39 (21.8)	0.99
Vomiting	47 (8.8)	14 (18)	24 (8.6)	9 (5)	0.003
Headache	88 (16.4)	15 (19.2)	52 (18.6)	21 (11.7)	0.11
Loss of smell	24 (4.5)	5 (6.4)	11 (4)	8 (4.5)	0.64
Loss of taste	24 (4.5)	6 (7.7)	11 (4)	7 (3.9)	0.33
Outcome of Covid-19					
Site of admission					
ICU admitted	81 (15.1)	11 (14.1)	40 (14.3)	30 (16.8)	0.75
Hospital	455 (84.9)	67 (85.9)	239 (85.7)	149 (83.24)	
Mortality					
In-hospital mortality	32 (6)	5 (6.4)	16 (5.7)	11 (6.2)	0.96
Recovered	504 (94)	73 (93.6)	263 (94.3)	168 (93.9)	

Moreover, characteristics such as diabetes (p=0.0001), hypertension (p=0.0001), chronic obstructive pulmonary disease (p=0.001), sex (male) (p=0.0003), and shortness of breath (p=0.018), were found to have an association with ICU admissions and in-hospital mortality (Table 2).

**Table 2:** Characteristics of COVID-19 patients according to outcome (ICU admission and in-hospital mortality). n (%) are reported unless otherwise stated. BMI: Body mass index, COPD: Chronic obstructive pulmonary disease

Characteristics	ICU-admitted (n=81)	Hospital (n=455)	P-value	In-hospital mortality (n=32)	Recovered (n=504)	P-value
Age (years), mean±sd	64.4±14.1	52.5±16.4	<0.0001	73.4±11.3	53.1±16.2	<0.0001
Sex (Male)	56 (19.6)		0.002*	27 (9.4)		0.0003
BMI (kg/m^2^), mean±sd	30.4±6.9	30.5±6.9	0.90	28.4±7.1	30.7±6.9	0.09
Co-morbidities	73 (18.7)	318 (81.3)	0.0002	32 (8.2)	359 (91.8)	<0.0001
Diabetes	53 (22)	188 (78)	<0.0001*	25 (10.4)	216 (89.6)	0.0001
Hypertension	53 (22)	188 (78)	<0.0001*	25 (10.4)	216 (89.6)	0.0001
Dyslipidemia	17 (14.5)	100 (85.5)	0.84	4 (3.4)	113 (96.6)	0.18
Asthma	6 (13.3)	39 (86.7)	0.72	5 (11.1)	40 (88.9)	0.12
COPD	2 (40)	3 (60)	0.11	2 (40)	3 (60)	0.001
Presence of symptoms	77 (16.04)	403 (83.96)	0.08	29 (6.04)	451 (93.96)	0.84
Cough	55 (16)	289 (84)	0.44	18 (5.2)	326 (94.8)	0.33
Fever	46 (15.7)	247 (84.3)	0.67	18 (6.1)	275 (93.9)	0.85
Sore throat	8 (9.4)	77 (90.6)	0.10	3 (3.5)	82 (96.5)	0.30
Shortness of breath	62 (23.9)	198 (76.2)	<0.0001*	22 (8.5)	238 (91.5)	0.018
Fatigue	21 (19.6)	86 (80.37)	0.14	6 (5.6)	101 (94.4)	0.85
Diarrhea	17 (14.7)	99 (85.3)	0.87	6 (5.2)	110 (94.8)	0.68
Vomiting	9 (19.2)	38 (80.9)	0.41	4 (8.5)	43 (91.5)	0.44
Headache	8 (9)	80 (90.9)	0.08	2 (2.3)	86 (97.7)	0.10
Loss of smell	1 (4.2)	23 (95.8)	0.12	0	24 (100)	-
Loss of taste	1 (4.2)	23 (95.8)	0.12	0	24 (100)	-

Table 3 presents the results of the multivariable regression analyses. Regarding the age, the unit odds ratios are reported, and these indicate the change in the odds of hospital admission to ICU admission and recovered status to death in hospital for every one-unit increase in the age variable. For example, in the outcome of in-hospital mortality, the unit odds ratio for age is 1.10. This value indicates that the odds of death are about 10% higher for every year the patient is older. The odds of ICU admission and in-hospital mortality are 2.30 and 5.4, respectively, for male patients compared to females.

**Table 3:** Association between the outcome of COVID-19 and patients’ characteristics using multivariate logistic regression analysis. ICU: Intensive care unit, BMI: Body mass index, OR: Odds ratio, CI: Confidence interval. Comorbidities and the presence of symptoms were not included in regression analysis for mortality related to COVID-19. Parameters were not estimable or unstable due to the number of patients in subgroups.

	ICU admitted		In-hospital mortality	
	OR (95% CI)	P-value	OR (95% CI)	P-value
Age, years	1.05 (1.03-1.10)	<0.0001	1.10 (1.07-1.14)	<0.0001
Sex (Male)	2.30 (1.34-3.95)	0.002	5.45 (1.91-15.49)	0.002
BMI, kg/m^2^	1.23 (0.96-1.04)	0.83	0.97 (0.91-1.04)	0.42
Vitamin D				
Deficient	1.08 (0.48-2.41)	0.80	1.74 (0.51-5.96)	0.46
Suboptimal	0.97 (0.92-0.56)	0.81	1.32 (0.55-3.19)	0.99
Co-morbidities	1.48 (0.61-3.55)	0.38	-	-
Presence of symptoms	2.44 (0.14-1.23)	0.11	-	-

## Discussion

Vitamin D has been known to have a role in promoting a better immune response against viral infections. Specifically, this study aimed to investigate the association between vitamin D deficiency and COVID-19 patients’ outcomes. Our findings suggest that patients who had vitamin D deficiency did not necessarily have worse outcomes when compared to patients with optimal vitamin D levels. Our findings suggest that ICU admissions and In-hospital mortalities were not associated with vitamin D deficiency. Among all patients, 81 were admitted to ICU. Out of whom, 13.6% were deficient, 49.4% had suboptimal levels, and about 37% had optimal levels of vitamin D. Our data also suggests that the association between vitamin D deficiency and occurrence of symptoms was statistically insignificant. Furthermore, the cough was the most prevalent symptom among all patients followed by fever and dyspnoea. Interestingly, vomiting was significantly associated with vitamin D deficiency. Another finding demonstrates that patients who developed dyspnoea were more prone to be admitted to the ICU. Additionally, analysis of comorbidities showed that hypertension and diabetes were associated with more ICU admission rates. Lastly, smoking was not associated with either ICU admissions or in-hospital mortality.

Many recent studies investigated the association between vitamin D deficiency and COVID-19 outcomes. Although our study revealed that vitamin D deficiency does not increase the severity of COVID-19, a meta-analysis and systemic review study concluded that vitamin D deficiency was associated with increased COVID-19 severity [16]. Another study conducted in Germany suggested that deficiency of vitamin D upon admission was associated with a higher incidence of invasive mechanical ventilation or death and worse survival [18]. Abrishami et al. support the previous studies by stating that low vitamin D serum levels were significantly associated with more pulmonary involvement and worse outcomes [19]. On the other hand, other studies that support our claim did not identify any association between vitamin D and outcomes of COVID-19. A study conducted in Saudi Arabia with 209 participants suggested that there was no association between vitamin D levels and COVID-19 severity [17]. Another cohort study done in Italy showed that low levels of vitamin D were not associated with a higher mortality rate [20]. Low levels of vitamin D in COVID-19 hospitalized patients were not associated with worse clinical outcomes [21]. Thus, the association between vitamin D deficiency and COVID-19 outcomes severity remains controversial.

Smoking, which is considered a risk factor contributing to the severity of COVID-19, was surprisingly found insignificant in one study upholding our finding [22]. The most common symptoms, according to a study done in Saudi Arabia, were reported to be cough, fever, fatigue, and shortness of breath which is consistent to some extent with our result [23]. Although most patients’ vaccination status was obtained, we could not reach out to 26 patients who were admitted to ICU or died; thus, we neglected their interpretation. While recent studies suggest that there is an association between vitamin D deficiency and the severity of COVID-19 outcomes, our findings reflect an opposite assumption. A reasonable explanation for the contrary finding is that our population in Saudi Arabia differs from other observed populations in those studies without considering ethnicity as a factor. Furthermore, only one study that supports our claim was done in Saudi Arabia. Moreover, the cut-off values defining vitamin D deficiency vary across studies which can alter group subdivision. One of our study strengths is that all individuals who met the study criteria were included. Therefore, the chances of bias in the sampling technique were avoided. Furthermore, the study criteria excluded patients who had factors that could affect their COVID-19 severity outcomes, which helped in determining the association between COIVD-19 and vitamin D. However, one of the study’s limitations is poor database documentation as many variables such as lab tests, vaccination status, smoking, and follow-ups at some points were missed or not well documented. In addition, vitamin D levels can be affected by chronic diseases which may subject the patients to higher COVID-19 severity.

## Conclusions

Our findings indicate there is no association between vitamin D deficiency and COVID-19 severity outcomes, and this could be attributable to various factors, such as the race of the patients. However, there were some symptoms that hold a higher percentage of occurrence in patients who are vitamin D deficient, such as vomiting. As the observational studies showed varying conclusions, future studies should be directed toward conducting RCT to determine whether vitamin D has an effective role in reducing COVID-19 severity.
